# A Mathematical Model of Levodopa Medication Effect on Basal Ganglia in Parkinson's Disease: An Application to the Alternate Finger Tapping Task

**DOI:** 10.3389/fnhum.2016.00280

**Published:** 2016-06-17

**Authors:** Chiara Baston, Manuela Contin, Giovanna Calandra Buonaura, Pietro Cortelli, Mauro Ursino

**Affiliations:** ^1^Department of Electrical, Electronic, and Information Engineering “Guglielmo Marconi,” University of BolognaBologna, Italy; ^2^IRCCS, Institute of Neurological Sciences of Bologna, Bellaria HospitalBologna, Italy; ^3^Department of Biomedical and Neuromotor Sciences, University of BolognaBologna, Italy

**Keywords:** mathematical model, neural network, Basal Ganglia, Levodopa, Parkinson's disease, finger tapping

## Abstract

Malfunctions in the neural circuitry of the basal ganglia (BG), induced by alterations in the dopaminergic system, are responsible for an array of motor disorders and milder cognitive issues in Parkinson's disease (PD). Recently Baston and Ursino (2015a) presented a new neuroscience mathematical model aimed at exploring the role of basal ganglia in action selection. The model is biologically inspired and reproduces the main BG structures and pathways, modeling explicitly both the dopaminergic and the cholinergic system. The present work aims at interfacing this neurocomputational model with a compartmental model of levodopa, to propose a general model of medicated Parkinson's disease. Levodopa effect on the striatum was simulated with a two-compartment model of pharmacokinetics in plasma joined with a motor effect compartment. The latter is characterized by the levodopa removal rate and by a sigmoidal relationship (Hill law) between concentration and effect. The main parameters of this relationship are saturation, steepness, and the half-maximum concentration. The effect of levodopa is then summed to a term representing the endogenous dopamine effect, and is used as an external input for the neurocomputation model; this allows both the temporal aspects of medication and the individual patient characteristics to be simulated. The frequency of alternate tapping is then used as the outcome of the whole model, to simulate effective clinical scores. Pharmacokinetic-pharmacodynamic modeling was preliminary performed on data of six patients with Parkinson's disease (both “stable” and “wearing-off” responders) after levodopa standardized oral dosing over 4 h. Results show that the model is able to reproduce the temporal profiles of levodopa in plasma and the finger tapping frequency in all patients, discriminating between different patterns of levodopa motor response. The more influential parameters are the Hill coefficient, related with the slope of the effect sigmoidal relationship, the drug concentration at half-maximum effect, and the drug removal rate from the effect compartment. The model can be of value to gain a deeper understanding on the pharmacokinetics and pharmacodynamics of the medication, and on the way dopamine is exploited in the neural circuitry of the basal ganglia in patients at different stages of the disease progression.

## Introduction

One of the main disabling features of Parkinson's disease (PD) is bradykinesia, defined as the progressive reduction in speed and/or amplitude of repetitive actions. Several rating scales are currently used to assess the clinical severity of PD, which include also behavioral tasks where the patient performs repetitive movements. Among the others, the finger tapping task (Picillo et al., 2016) is one of the simplest, being able to provide information about the severity of bradykinesia. In particular, frequency of alternate finger tapping on two separate keys has been shown to correlate strongly with part III of the Unified Parkinson's Disease rating scale (UPDRS III; Fahn and Elton, 1987), namely with the bradykinesia subscore (Homann et al., 2000; Taylor Tavares et al., 2005) and to have the highest sensitivity to discriminate PD patients from the general population (Pal et al., 2001).

The usefulness of the alternate finger tapping task for specific PD motor assessment has been proven by evidences showing correlation with the extent of loss of neurons in the substantia nigra, assessed *in vivo* with [^18^F]-6-fluoro-L-dopa (6-FD) PET (Pal et al., 2001). The correlation just mentioned is considered relevant since the substantia nigra is the brain structure of the basal ganglia (BG) in which the death of dopaminergic neurons is responsible of the major motor symptoms of the disease.

At present, neither pure causal treatment nor neuroprotective mechanisms are available for PD. As previously reported (Contin and Martinelli, 2010) alternate finger tapping test seems appropriate for the evaluation of levodopa (LD) effects on bradykinesia, proving to be consistently affected in PD subjects compared with a control group and a sensitive and reproducible indicator of drug effect. As the disease advances, the practical benefits of levodopa are hindered by modifications in drug kinetic and dynamic mechanisms, resulting in a fluctuating response during the day. Oral doses of levodopa at first achieve a “long duration effect” persisting longer than the plasma half-life of the drug, but with the progression of the disease the clinical response becomes more dependent upon the rise and fall of plasma LD concentrations. No differences in LD pharmacokinetics have been observed in the shift from a “stable” to a “fluctuating” response to LD doses (Contin et al., 2001).

The relevance of the information provided by the finger tapping task, and its association with the pattern of the response to levodopa, is usually assessed with empirical equations, which assume a non-linear relationship (similar to the Hill law) between levodopa concentration in the brain and the frequency of tapping (Sheiner et al., 1979; Contin et al., 2001; Chan et al., 2004). Of course, this information is incomplete and risks of being under-utilized if not related with the neural circuitry of the basal ganglia, directly involved with the disease and responsible for movement's initiation and termination. In particular, it is well known that the response to levodopa affects the balance between Go (direct) and No Go (indirect) circuitry in the BG (Albin et al., 1989; Frank, 2005; Schroll and Hamker, 2013) which, in turn, results in the observed bradykinesia and the appearance of motor fluctuations. Indeed, the key point to understand the symptoms of PD, and their temporal deterioration, should be searched in the relationship between the activity of neurons in the BG (especially in the striatum) and dopamine (or levodopa) levels in the brain.

Neurocomputational models, inspired by biology, represent a powerful tool to quantify the main mechanisms involved in a complex neural system, and to relate behavioral patterns with the underlying neural circuitry. These models can also mimic the plastic changes induced by experience (such as the effect of reward and punishments on synapse potentiation and depotentiation) and the role of tonic and phasic alterations in neurotransmitter levels. The past years have seen a richness of neurocomputational models of the BG, with the emphasis on different neurophysiological or clinical problems (Frank, 2005; Wiecki and Frank, 2010; Schroll et al., 2012; Helie et al., 2013). Recently, we developed a neural model of the BG (Baston and Ursino, 2015a), which represents a good compromise between simplicity and accuracy. The model includes the three main routes operating in the BG circuitry (that is, the direct (Go), indirect (No Go), and hyperdirect pathways). Furthermore, it incorporates the role of dopamine (both tonic and phasic, i.e., dopamine peaks or dips during reward and punishment), synapse plasticity, and the role of the cholinergic interneurons (affected by dopamine levels themselves). Preliminary simulations performed in conditions of altered dopamine (Baston and Ursino, 2015b) show that, in the model, the time required to accomplish an action crucially depends on the tonic dopamine level, in a way coherent with the present knowledge of PD symptoms. Hence, we claim this model may be a suitable innovative tool to simulate bradykinesia (and in particular the tapping frequency) by relating the neural mechanisms with dopamine/levodopa levels in the brain.

Accordingly, the aim of this work is to quantify the connection between levodopa levels and finger tapping performances by means of biologically inspired models and computer simulations. In particular, we want to show that the model is even able to reproduce the qualitatively different finger tapping pattern in time of PD patients with no motor fluctuations (here referred as group 1) and PD patients showing motor fluctuations (referred as group 2). With this objective in mind, the neurocomputational BG model has been linked with a classic model of levodopa pharmacokinetics and pharmacodynamics. In particular, the BG model used has the same structure as the model presented in Baston and Ursino (2015a) including the main neural structures (cortex, Go, and NoGo neurons in the striatum, subthalamic nucleus, globus pallidus pars interna and externa, thalamus) involved in action selection by the BG. The difference is that here we assumed only two possible actions to be selected, and we simulated a dynamic shift between these actions to mimic an alternate finger tapping task. In other words, the model is used to perform a dynamic task; conversely, in our previous works, the model was used to perform a static task, in which just a single static choice had to be selected. The global model aspires to simulate the entire chain of mechanisms involved in the patient behavior, from levodopa administration to the response of motor cortical neurons.

With the model, we first analyzed how different dopamine levels may affect the frequency of tapping. Then, we simulated the temporal patterns of levodopa concentration in plasma and the temporal pattern of tapping frequency in six PD patients, during 4 h after levodopa administration.

## Materials

### Patients

We retrospectively modeled levodopa (LD) test results obtained from six PD patients referred to the Institute of Neurological Sciences for therapeutic drug monitoring (TDM; Contin et al., 2001). Patients had given their written informed consent to personal data processing for research purposes. These patients were divided in two groups, on the basis of motor fluctuations: group 1 included patients without motor fluctuations, and group 2 patients showing motor fluctuations.

Clinical characteristics of patients are reported in Table 1.

**Table 1:** **Clinical characteristics of each patient: age [years], Parkinson's disease symptom duration [years], levodopa therapy duration [years], anti-parkinsonian cotherapy dose (PRA stands for pramipexole, ROP for ropinirole and RAS for rasagiline) [mg/day], levodopa dose per day [mg/day], Unified Parkinson's disease Rating Scale (UPDRS) III scores, Hoehn and Yahr scores**.

**Group**	**Subject**	**Age**	**Sex**	**PD symptom duration**	**LD therapy duration**	**Anti-PD cotherapy dose**	**LD dose**	**UPDRS III**	**H and Y**
1	1	78	m	3	0.5	–	300	11	2
	2	59	m	2	0.5	–	200	11	1
	3	62	f	3	1	PRA–2.1	400	27	3
2	1	65	m	6	5	PRA–0.26	400	18	2
	2	52	f	5	2.5	–	300	21	2
	3	56	f	4	3.5	ROP–6.0 RAS–1.0	450	29	2

### Levodopa kinetic-dynamic test

On the morning of TDM the patients received an oral fasting dose of LD/benserazide (100/25 mg) after a 12 h washout of LD.

Blood venous samples (2 ml) for measurements of plasma LD concentrations were drawn by an indwelling catheter immediately before the drug dose, at 15 min intervals for the first 90 min, then on the half hour up to 3 h after dosing. Blood specimens were collected and processed for plasma LD analysis as reported previously (Baruzzi et al., 1986).

Patients' motor response to the LD test dose was assessed by the alternate finger tapping test simultaneously with blood sample collection (Contin et al., 2001) up to 4 h post-dosing. This test objectively measured the number of times the patient could alternately tap two buttons 20 cm apart in 60 s with the most affected hand, using a touch-screen computerized system. Patients were comfortably seated in an armless chair and instructed to alternate tap the two touch-sensitive buttons as fast and as accurately as possible.

Latency to onset of a clinical significant motor response elicited by the LD test dose is defined as the time to increase in tapping frequency of ≥15% of baseline values. Duration of the tapping effect was calculated as the difference between the time to return to <15% of baseline values and time to onset of response.

For the objective of the present modeling application, patients were defined “stable” or “wearing-off” responders when no return or return to baseline tapping performances, respectively, was observed within the 4 h length of examination.

## Model description

The overall model consists of:

(i) a two-compartment description of pharmacokinetics;
(ii) a single compartment effect description, with a non-linear sigmoidal relationship between concentration and the effect;
(iii) a neurocomputational model of the basal ganglia, which converts the effect (dopamine + levodopa) into action selection. In this particular work, the BG accomplish an alternate movement of one finger, from which the tapping frequency is computed.

**Figure 1:**
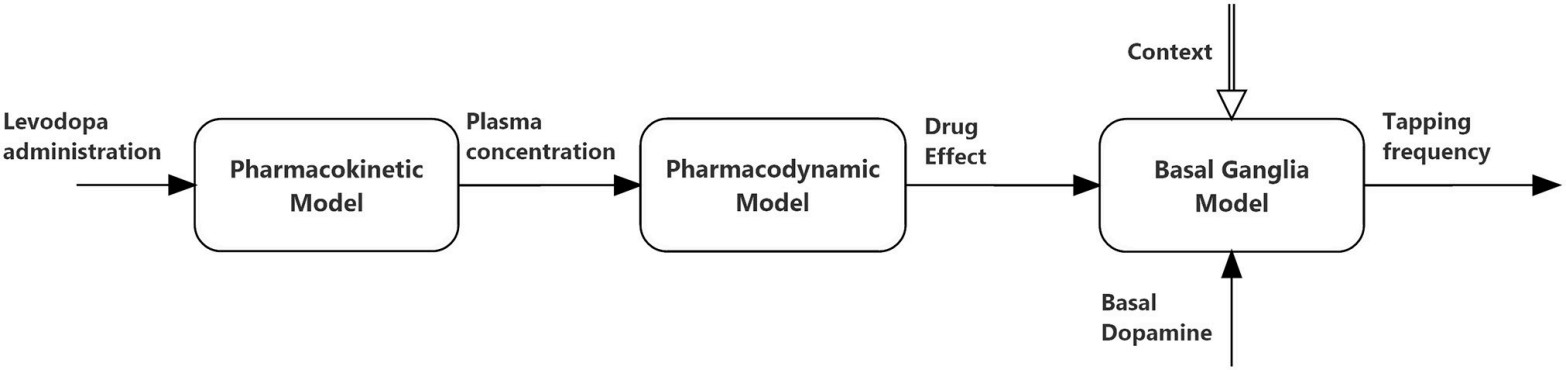
**Block diagram describing the connections between the three sub-models (pharmacokinetics, pharmacodynamics, and basal ganglia) used in the present simulations**.

The relationships between the different parts of the global model are depicted in Figure 1, and illustrated qualitatively below. Equations can be found both in the main body of the manuscript and in the Appendix in Supplementary Material.

### Modeling the levodopa pharmacokinetics

**Figure 2:**
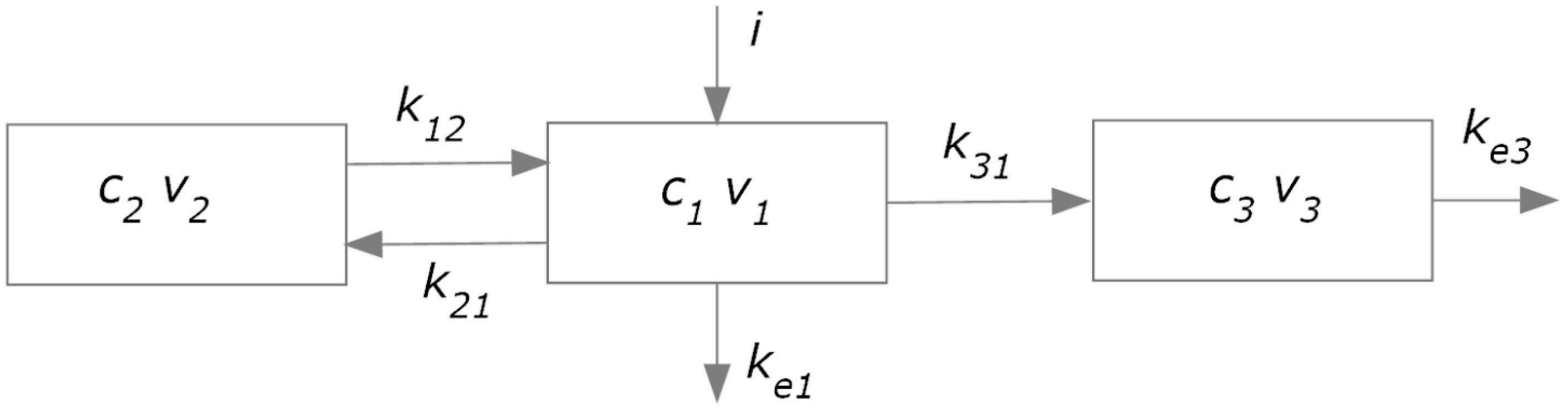
**Compartment model used to simulate plasma pharmacokinetics (compartments 1 and 2) and the drug effect in the brain (compartment 3)**.

The kinetics of levodopa was simulated using an approach similar to that used in former papers (Sheiner et al., 1979; Contin et al., 2001; Chan et al., 2004). The overall model (subdivided in a plasma model and an effect compartment) is illustrated in Figure 2.

A two-compartment model (blocks 1 and 2 in Figure 2) was adopted to describe plasma levodopa concentration. This corresponds to the following equations:

(1) $$\begin{array}{rcl} V_{1}\frac{dc_{1}}{dt} & = & -\left(k_{21}+k_{31}+k_{e1}\right)c_{1}+k_{12}c_{2}+i \end{array}$$

(2) $$\begin{array}{rcl} V_{2}\frac{dc_{2}}{dt} & = & k_{21}c_{1}-k_{12}c_{2} \end{array}$$

The first represents a central compartment, where levodopa is administered and plasma concentration is measured. The second is a peripheral compartment, representing the interaction between plasma and other body fluids. As shown in Figure 1 and in the equations, the model contains five parameters: the inter-compartment rate constants (*k*_12_ and *k*_21_), the total body rate constant (*k*_*etot*_ = *k*_*e*1_+*k*_31_), and the compartment volumes (*V*_1_ and *V*_2_). It is worth noting that, compared with some previous models (Chan et al., 2004, for instance) we adopted two different values for the inter-compartment rate constants. This choice is necessary to obtain good fitting of the measured plasma concentration values (see Section Results). Assuming the same rate (i.e., *k*_12_ = *k*_21_) results in poor fitting of real data.

The parameters describing the levodopa plasma kinetics were estimated in individual patients by minimizing a least-square criterion function of the difference between model predictions and *in vivo* data of plasma concentration, during 4 h after levodopa administration. Minimization was achieved using the Nelder-Mead algorithm (Press et al., 2007), which performs a direct search in the parameter space and does not require the computation of gradient. However, in order to reduce the number of estimated parameters (thus reducing the risk of overfitting) we maintained *V*_1_ and *V*_2_ at constant values, and only parameters *k*_12_, *k*_21_, and *k*_*etot*_ were estimated individually. The fixed values of *V*_1_ and *V*_2_ were mean values taken from Chan et al. (2004), who essentially considered a subject with a standard weight of 70 kg. A personalization on the individual weights may be adopted in future works; however, we deem this specialization uninfluential at present, since our aim was to achieve a good fitting of plasma concentration, to be used for the downstream effect compartment.

The input to the central compartment, representing the levodopa administration, was maintained constant during a certain period, to mimic a progressive oral assimilation. The duration of this period corresponds to the period in which the measured plasma levodopa concentration increased progressively. The constant value was computed so that the overall amount of administered levodopa was equal to the experimental one.

### Modeling the levodopa pharmacodynamics

In order to simulate the effect of levodopa on the basal ganglia, and therefore on the finger tapping response, we needed to calculate the levodopa concentration in the “effect compartment.” We used the model proposed by Sheiner et al. (1979); this model assumes that the drug concentration in plasma and in the effect compartment tends to become proportional in steady state conditions (i.e., when all transient phenomena have exhausted, and all quantities settle at a constant equilibrium value), and that no levodopa comes back from the effect compartment to the central one. Hence, thanks to the last assumption, the effect compartment does not affect the kinetics of the plasma compartment, provided a single parameter (*k*_*etot*_) is used to describe total body clearance. As shown in Figure 2, the effect compartment contains three parameters: *k*_31_, *k*_*e*3_, and *V*_3_, which describe the drug absorption from the central to the effect compartment, the drug removal from the effect compartment, and the compartment volume, respectively. However, these parameters are not independent, since a combination of them produces the same model output. First, only two parameters actually appears in the equations, i.e., *k*_31_∕*V*_3_ and *k*_*e*3_∕*V*_3_. Furthermore, it can be demonstrated that the shape of the *c*_3_ (the concentration of levodopa in the effect compartment) temporal pattern depends only on the ratio *k*_31_∕*V*_3_. In fact, the general solution of Equation (3):

(3) $$\begin{array}{l} V_{3}\frac{dc_{3}}{dt}=k_{31}c_{1}-k_{e3}c_{3} \end{array}$$

can be written as follows (assuming no levodopa in the effect compartment at the instant *t* = 0):

(4) $$\begin{array}{l} c_{3}\left(t\right)=\frac{k_{31}}{V_{3}}\int_{0}^{t}c_{1}\left(\tau \right)e^{-\frac{k_{e3}}{V_{3}}\left(t-\tau \right)}d\tau \end{array}$$

Hence, the ratio *k*_31_∕*V*_3_ represents only a proportionality factor for the previous equation. This can be accounted for by a different value of parameter *D*_*c*50_ in the subsequent equation (Hill law, law, Equation (6)), without affecting the overall fitting procedure. Therefore, without a loss of generality, we used a fixed value for the parameter *k*31/*V*3 in all trials, and only the remaining parameter *k*_*e*3_/*V*_3_ was assigned individually. This parameter was estimated, together with the other parameters describing the pharmacodynamics, by fitting the global model to the finger tapping frequency values (see below).

Finally, we needed a law describing how the concentration in the effect compartment (i.e., *c*_3_) affected the activity of the neurons in the striatum. In fact, as described in the sub-section “Modeling the basal ganglia” below, our neurocomputational model assumes an input quantity (named *D*) representing how dopamine modulates the activity of the Go and NoGo neurons.

First, in order to account for the observed delay between plasma concentration and the clinical response, we introduced a pure delay (say *T*) between the computed concentration in the effect compartment and its action on the striatum neurons. The delayed concentration will be named *c*_3*delay*_ below. We have:

(5) $$c_{3delay}\left(t\right)=\;c_{3}\left(t-T\right)$$

A classic way to describe the binding of a molecule with a receptor (or a reaction with cooperative effects, where an enzyme can bind one or more substrate molecules) is the Hill law (Keener and Sneyd, 2009). We can write:

(6) $$D=D_{0}+\frac{D_{max}c_{3delay}^{N}}{D_{c50}^{N}+c_{3delay}^{N}}$$

where *D*_0_ represents the basal value (i.e., the effect immediately before the beginning of levodopa administration) and the second term, with a sigmoidal shape, represents the effect induced by a levodopa concentration *c*_3_, delayed by the time *T*. *D*_*max*_ is the maximum effect that levodopa can produce, *D*_*c*50_ is the levodopa concentration which produces 50% of the maximum effect, and *N* is the Hill coefficient, which determines the slope of the concentration-effect relationship.

All the parameters in the previous equation (*D*_0_, *D*_*max*_, *D*_*c*50_, and *N*) together with the parameter *k*_*e*3_ in the effect compartment, and the delay *T*, were assigned to simulate the tapping frequency values measured on patients during 4 h after levodopa administration. At present, the estimation was performed manually, through trial and error adjustments of the parameters. The reason for this choice is that the tapping frequency exhibits significant outliers, which preclude a reliable automatic fitting. An automatic better fitting will be attempted in future works. Indeed, the aim of this preliminary work was not to achieve parameter estimation automatically, but rather to show that the present neurocomputational model of the BG can simulate the behavior of disparate patients, at different stages of PD severity.

### Modeling the basal ganglia

A significant difference of this model compared with previous ones (for instance, Contin et al., 2001; Chan et al., 2004) is that Equation (6) was not used to fit the tapping frequency directly, but is used as an external input for the neural network model described below. The advantage is that, after parameter estimation, the model may be used to simulate other tests in the same patients, or to make additional predictions on the patient behavior, thus providing a much more flexible interpretation of the neurological status (see Section Discussion). We are aware that different parts of the basal ganglia may have different functions (for instance cognitive in the basal part and motor in the dorsal part) and that these may be differently damaged in different patients. Since we simulate a motor test (the finger tapping) we can assume that the model, and the estimated parameters, refer to the dorsal portion of the BG.

**Figure 3:**
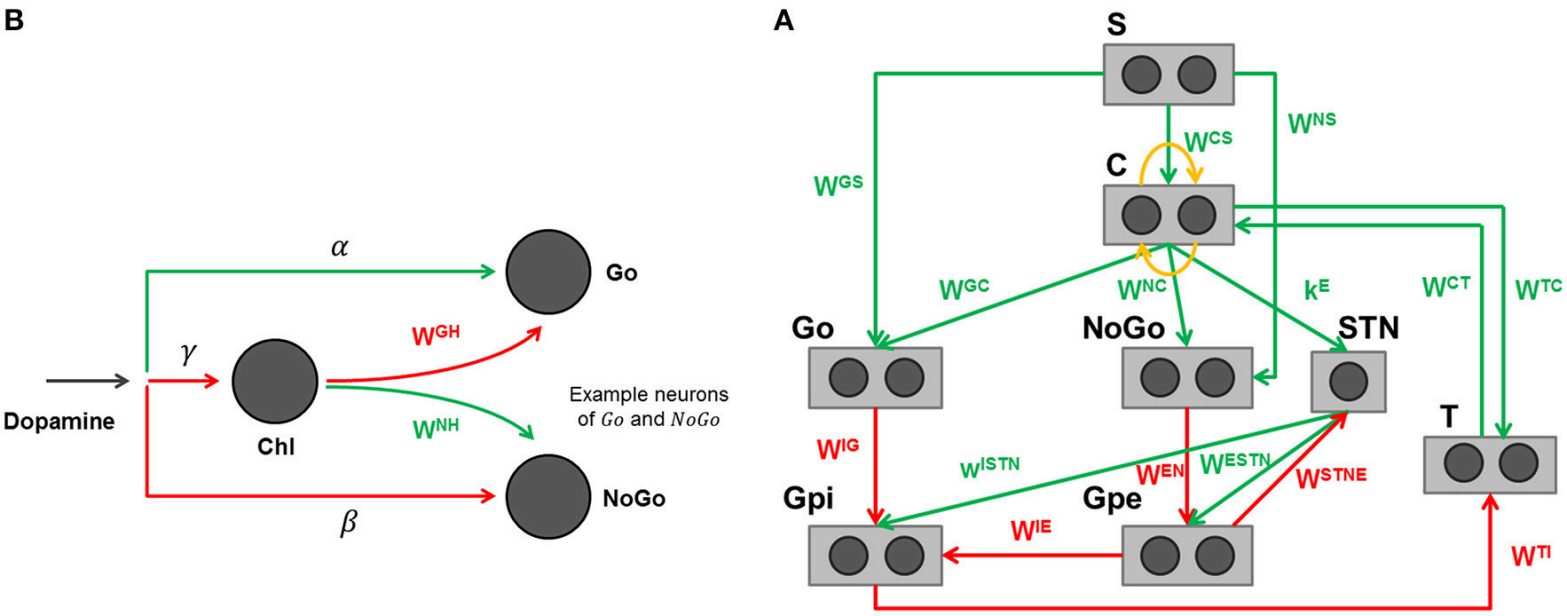
**(A)** Graphical representation of the basal ganglia model adapted to reproduce the finger tapping task. Rectangles represent different structures, circles neurons, arrows projections: green excitatory, red inhibitory, orange lateral inhibition. **(B)** Effect of dopamine and cholinergic interneuron on Go and NoGo cells in the model. Arrows projections: green excitatory, red inhibitory.

The specific structure of the network is depicted in Figure 3A, with further details clarified in Figure 3B. The detailed description of the BG computational model, including its equations, is presented in the Appendix in Supplementary Material. Parameters and synaptic weights of these equations are reported respectively in Table 4 and Table 5.

Each neuron in the model is represented as a computational unit, which calculates its activity from the weighted sum of inputs. The output activity of each neuron is in the range [0, 1], representing a normalized firing rate. In particular, we used a sigmoidal static relationship to represent the presence of a lower threshold and upper saturation for neuronal activity, and a first order low-pass dynamics to mimic the integrative capacity of neuron membrane.

The model includes a sensory representation (S), which represents the external context, and the corresponding motor representation in the cortex (C). This representation considers several actions in mutual competition, each represented by a segregated channel. In the previous model version (Baston and Ursino, 2015a,b) we assumed four actions for simplicity. In the present work, devoted to a simulation of the alternate finger tapping test, only two actions are considered and sufficient to represent the task in an adequate way: tapping down (action 1) and lifting the same finger up while moving it to the other position (action 2). For this reason, just two different and segregated action channels are shown in Figure 3A, each coding for a different alternative choice. Of course, including more channels, the network can also be used to simulate more complex alternative choices.

Moreover, the model includes the thalamus (T), the striatum, functionally divided according to dopamine (DA) receptor expression (D1: Go or G, D2: NoGo or N), the subthalamic nucleus (STN), the globus pallidus pars externa (Gpe or E) and an output part represented by the globus pallidus pars interna (Gpi or I) and the substantia nigra pars reticulata (SNr) taken together. This framework implements the three main pathways (direct, indirect, and hyperdirect; Albin et al., 1989; Nambu et al., 2002) used by the BG. In absence of sufficient stimuli, all actions are inhibited, due to a prevalence of the No Go pathway on the Go one, which leads to inhibition of the thalamus. In the presence of a sufficient external stimulus, the cortex can select a response based on a competition among cortical neurons. This competition is realized with a winner-takes-all (WTA) process, implemented via lateral inhibition in the cortex and a positive feedback from the thalamus. In particular, if the Go pathway prevails on the No Go within an action channel, the corresponding neuron in the thalamus is disinhibited, thus providing an excitatory input to the corresponding cortical neuron. This positive loop maintains the winner neuron to a high level (the action is selected) while the other rival neurons in the cortex are inhibited.

A simplification in the model consists in the use of the dopamine effect (*D*) directly as a modulating input factor, without explicitly representing the dopaminergic neurons in the substantia nigra pars compacta, which are responsible for the release of the dopaminergic neurotransmitter. This choice allows simple simulations of normal and pathological conditions, in which dopamine levels can be artificially altered by the disease or by external intervention. It is worth noting that *D* does not represent the real tonic dopamine level, but an input that modulates the working point of the Go and NoGo neurons in the striatum. High values of *D* mean high dopamine effect on receptors in the striatum. Conversely, low values of *D* indicate a poor dopamine effect. Experimental studies show that dopamine can exert different effects depending on the receptor (Hernández-López et al., 1997; Hernandez-Lopez et al., 2000). In particular, the effect of *D* is different within the striatum, being primarily excitatory for the Go part and inhibitory for the NoGo part. As a result, high values of *D* favor a rapid selection of actions, whereas low values of *D* are associated with a prevalence of the No Go, and so with slow or inhibited actions. Furthermore, we also included a contrast enhancement effect on the Go neurons (Frank, 2005), i.e., the quantity *D* is able to excite only the neurons in the Go pathway with a high excitation, thus further potentiating their response, but has an inhibitory effect on Go neurons with poor excitation. This mechanisms further help the WTA dynamics, accelerating the choice of a winner action.

A novelty of this model, presented for the first time in Baston and Ursino (2015a), is the description of the cholinergic pathway. Indeed, dopamine exerts its effect on the striatum not only directly (i.e., by exciting Go neurons with high activity and depressing both Go neurons with poor activity and NoGo neurons) but it also inhibits the cholinergic pathway (see Figure 3B). The latter, in turn, has an opposite effect on the striatum, favoring the No Go pathway, and depressing the Go pathway. Since the cholinergic system is active at rest, and is inhibited by a dopamine increase, the two mechanisms work in synergy. In particular, we observed that a change in dopamine level *per se* is insufficient to have appropriate responses, without the potentiation induced by the synergistic cholinergic effect. A lesion of the cholinergic mechanisms in the model would reduce the dopaminergic influence, thus resulting in a further bradykinesia.

Finally, evidences (Schultz, 1998) show that BG are able to modify their synaptic weights, in particular those entering into the Go and NoGo striatal neurons. This relies on dopamine and acetylcholine (Ach) changes. In particular, plasticity occurs in case of punishment or reward, when phasic changes in dopamine (a transient peak during rewards; a transient dip during punishments) induce a synaptic change via Hebbian mechanisms. A dopamine peak, with the consequent fall in Ach, further excites the winner Go neurons, and depresses the NoGo neurons, thus causing Hebbian potentiation of the winning action and depotentiation of all other actions. The opposite effect occurs during punishment.

In this study, however, differently from our previous work, we only test different tonic dopamine levels, avoiding the analysis of phasic changes and neglecting possible synapse plasticity during the trials.

In conclusion, the BG, through the combined action of the three pathways described above, modulate the inhibition provided from the Gpi to the thalamus, thus consenting or blocking the WTA process. Ultimately, it is the imbalance between the two pathways (Go and No Go), due to different values of the synapses and of dopamine level, that modulates the activity of the Gpi. If the Go pathway prevails, the Gpi provides less inhibition to the corresponding neuron of the thalamus (i.e., the BG “let go” the response). On the contrary, if the No Go pathway is more active, the Gpi provides more inhibition to the thalamus (i.e., the BG “stop” the response). Challenging situations, characterized by a high conflict among alternative actions, are managed by the hyperdirect pathway, carried out by the STN: its role is to provide an overall stop signal to all the units of the Gpi in order to prevent many simultaneous cortical winners and let the cortex more time to solve the conflict.

## Results

In the first part of this section, we will present some computer simulations results, to show how the model can simulate the alternate finger tapping test, and how the pattern of cortical motor neurons reflects the level of tonic dopamine.

In the second part, we will present the results of parameter estimation, obtained by fitting the overall model (pharmacokinetic-pharmacodynamic and neurocomputational) to the data obtained on six patients.

### Simulation of a finger tapping test

**Figure 4:**
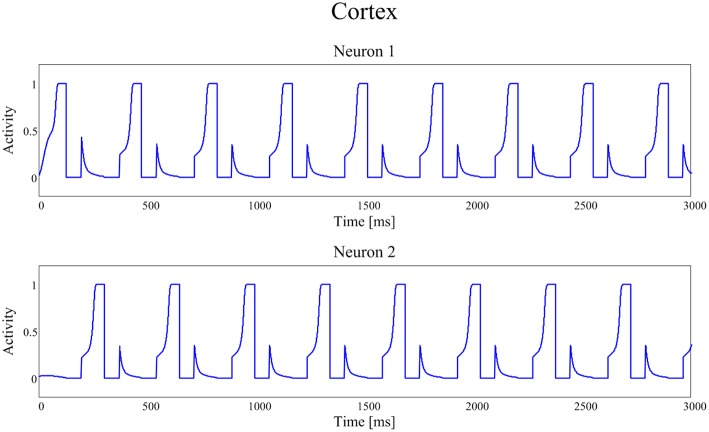
**Temporal patterns of neural activities of the first neuron (top panel) and of the second neuron (bottom panel) of the cortex C, when the dopaminergic input is typical of an healthy subject (frequency *f* = 2.89 Hz (173 taps/min))**.

**Figure 5:**
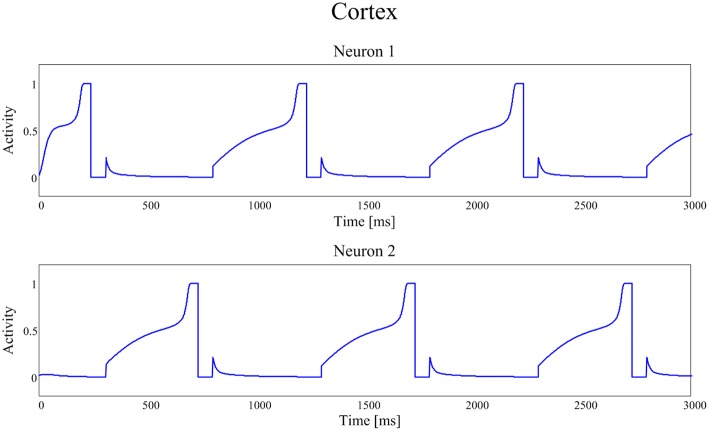
**Temporal patterns of neural activities of the first neuron (top panel) and of the second neuron (bottom panel) of the cortex C, with a dopamine input typical of a PD patient (frequency *f* = 1.00 Hz (60 taps/min))**.

Figures 4, 5 show the temporal pattern of activity in the two cortical motor neurons, during two different simulations. We assume that the first neuron of the cortex (top panel of Figures 4, 5) encodes the tapping, i.e., the movement downwards of the finger in either position, while the activity of the second neuron of the cortex (bottom panel of Figures 4, 5) encodes its movement upwards together with the spatial shift. The first simulation was performed using a high value of *D*, typical of an healthy individual. The second was performed using a low value of the parameter *D*, typical of a PD patient.

In our model the external sensory input is used to denote the action to be selected (1 means that the action is strongly preferred, 0 no preference for this action). During these simulations we first presented a stimulus S = [1 0], which means excitation to the first channel and inhibition to the second. This provokes the activation of the first channel, inducing the finger to go down in the first position, preventing at the same time its movement upwards (deactivation of the second channel). However, for the movement to initiate, we need the Go pathway to prevails on the No Go one, to activate the thalamus and to allow the corresponding cortical neural activity to reach a level close to 1. In fact, we assumed that an action starts only when the cortical activity exceeds a given threshold, close to the maximum. This requires a transient period, clearly evident in Figures 4, 5.

Furthermore, we assumed that, when an action has been selected, the input stimulus is reversed (in the previous example, we now provide an input S = [0 1] which excites the second action channel) to represent that the subject is now trying to perform the second action, which induces the movement of the finger upward and the shift toward the second position. A physiological time delay of 100 ms has been included after the beginning of the first action, to account for the physiological time necessary to initiate the movement, detect the action and communicate it to the central neural system. Similarly but opposite to the previous case, this time the finger has to lift up and shift, avoiding the tapping down. Again, after a transient period, the activity of the second neuron of C reaches its action threshold level, inducing the lifting of the finger and a new reverse of the input stimuli.

We are aware that simulating the tapping task as a simple choice between two actions (finger down in either position, finger up and shift) is a strong simplification. Of course, the real movements consist of a sequence of simpler movements, suitably chained. We summarized this chain of movements using only two macroscopic choices to reduce model complexity to a minimum. Moreover, with this assumption we are able to fully reproduce clinical data of the finger tapping task.

The activities of the two neurons of the cortex follow this iterative pattern, establishing recurring alternate signals.

In Figure 4, parameter *D* is 0.55 and the frequency of the neural signals is 2.89 Hz (173 taps/min), reflecting the short time required for the winner neuron to reach the action threshold.

Figure 5 shows the same recurring alternate pattern for cortical activities, but with a tonic *D* = 0.22. Now the frequency of the neural signals is just 1.00 Hz (60 taps/min), indicating a severe stage of bradykinesia. In this last case it is worth noting the very long time required for the winner neuron to reach the threshold level that initiates the action: this is the consequence of the low levels of the input *D*, which inhibit the Go neurons and potentiate the NoGo ones.

Iterating the previous procedure several times, i.e., establishing a level of tonic *D* and evaluating the frequency of the neural activities patterns of the cortex (and so the alternate finger tapping task), a curve that maps *D* into the tapping frequency, and therefore tapping score, can be built.

**Figure 6:**
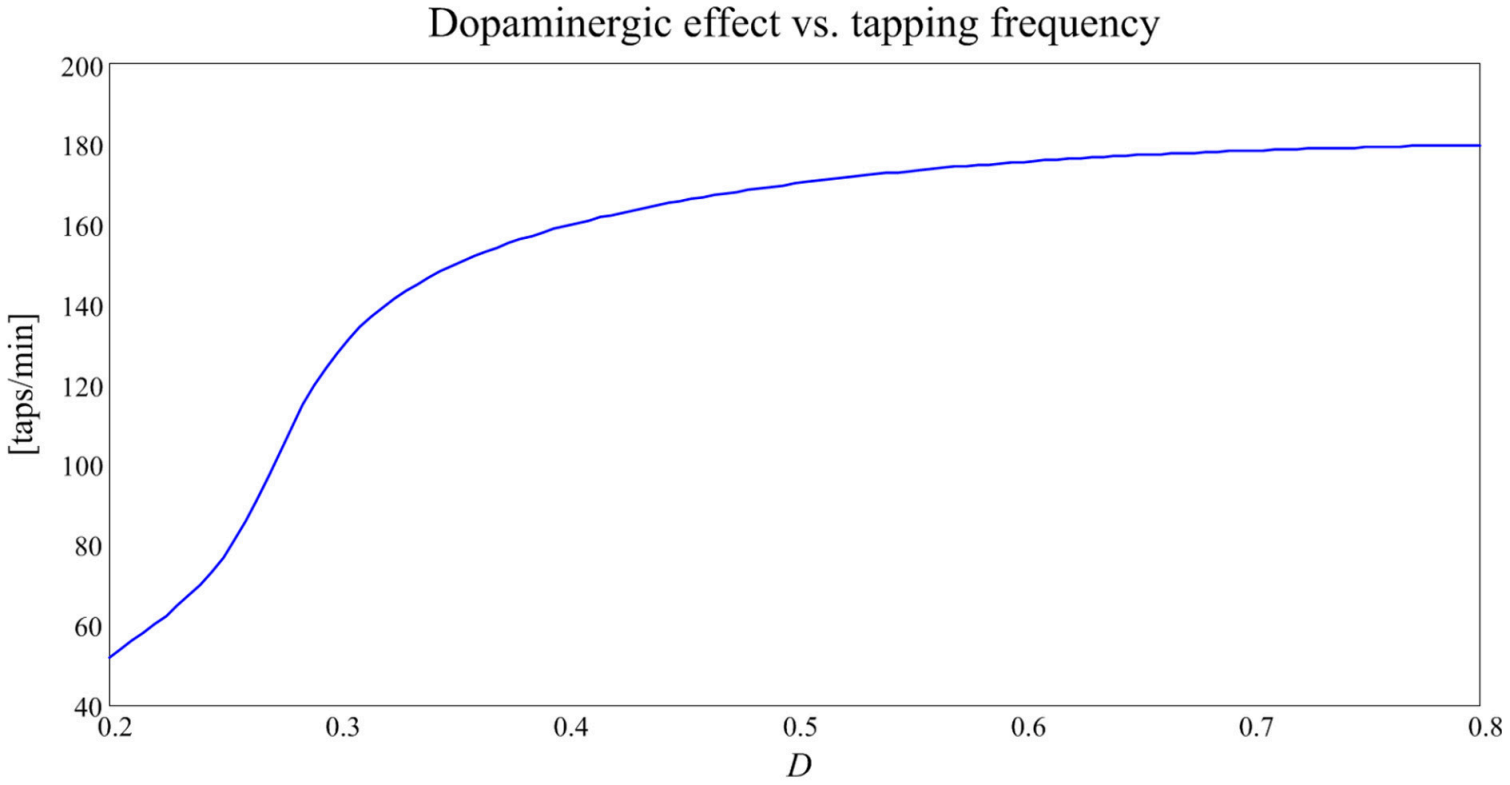
**Curve expressing the relationship between the dopaminergic input, *D*, and tapping frequency [taps/min] in the alternate finger tapping task**.

As it is evident from Figure 6, this curve has a clear monotonic trend, with an upper saturation. An increase in *D* corresponds to an increase in the tapping frequency, more pronounced for lower values of *D* and nearly negligible for higher values of *D*, where the curve reaches saturation.

In our model, tonic *D*-values typical of Parkinson's disease may be between 0.2 and 0.3; in this range the curve obtained predicts that the tapping frequency for PD subject should be between 1 and 2 Hz. On the contrary, tonic *D*-values typical of healthy subjects in our model are set between 0.4 and 0.5, and the same curve predicts that the tapping frequency should be around 3 Hz. In other words, PD subjects are slower at performing the finger tapping task. This finding is coherent with the well-known bradykinesia of PD subjects compared to the normal motor behavior of healthy subjects (Contin et al., 1990).

### Parameter estimation in PD patients

As previously specified, the patients were subdivided in two groups, on the basis of the duration of the tapping response. Parameters describing the pharmacokinetics (*k*_12_, *k*_21_, and *k*_*etot*_) were fitted on the plasma concentration curves, while the other parameters (*k*_*e*3_∕*V*_3_, *T*, *D*_0_, *D*_*max*_, *D*_*c*50_, and *N*) were assigned to simulate the finger tapping frequency.

**Figure 7:**
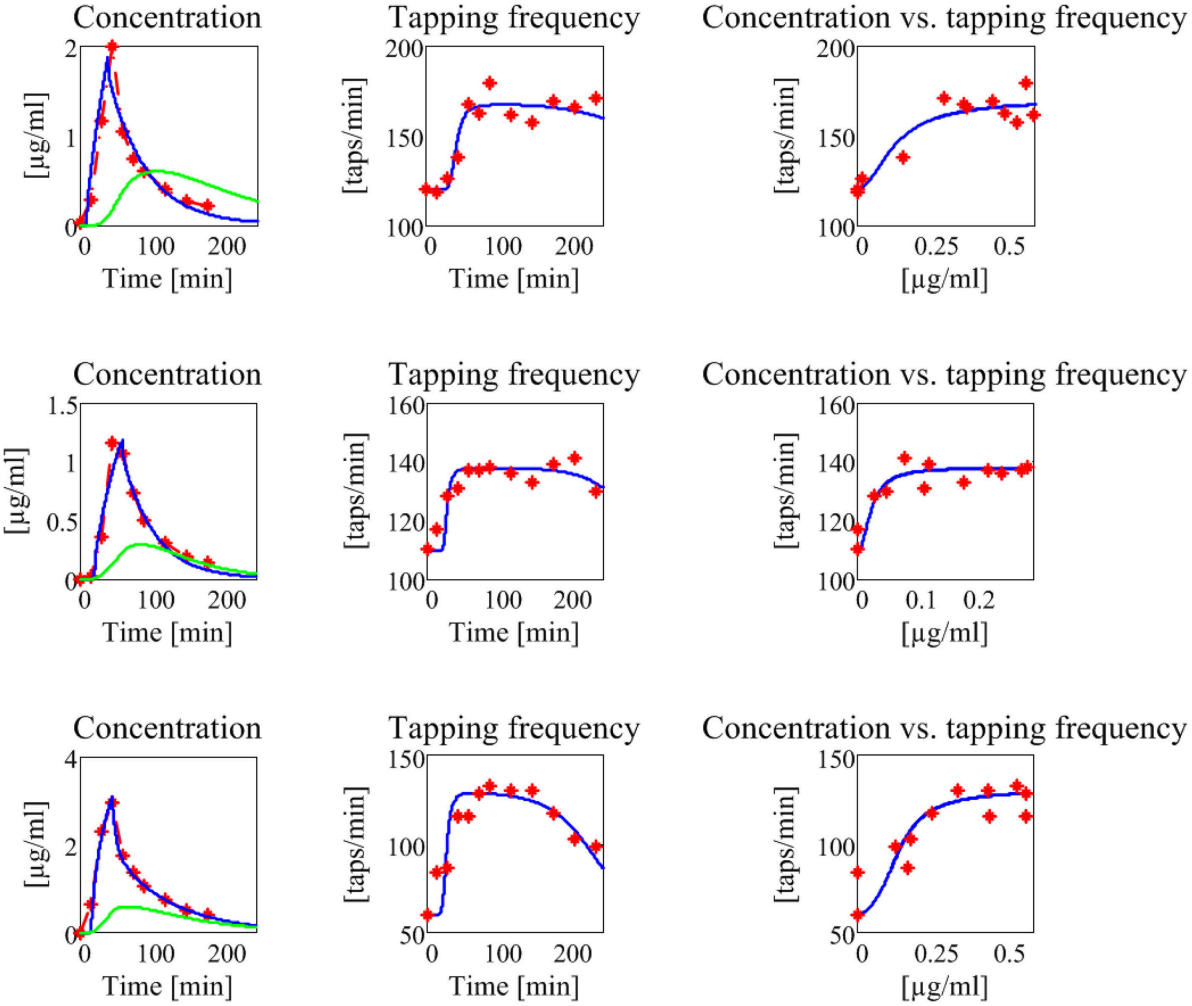
**Results concerning the three patients in the first group (subjects with no motor fluctuations)**. The first line represents the subject 1, the second the subject 2, and the third the subject 3. The first column reports the experimental data of plasma levodopa concentration (red dot and dashed line), the corresponding fitting curve (blue line) and the delayed estimated brain levodopa concentration curve (green line) vs. time. All concentrations are represented in μg/ml. The second column represents the tapping frequency [taps/min] vs. time [min]. Experimental data are represented with red dots, the fitting curve with a blue line. The third column represents the tapping frequency [taps/min] plotted vs. brain levodopa concentration [μg/ml]: experimental data are represented with red full points, estimated values with a blue line.

**Figure 8:**
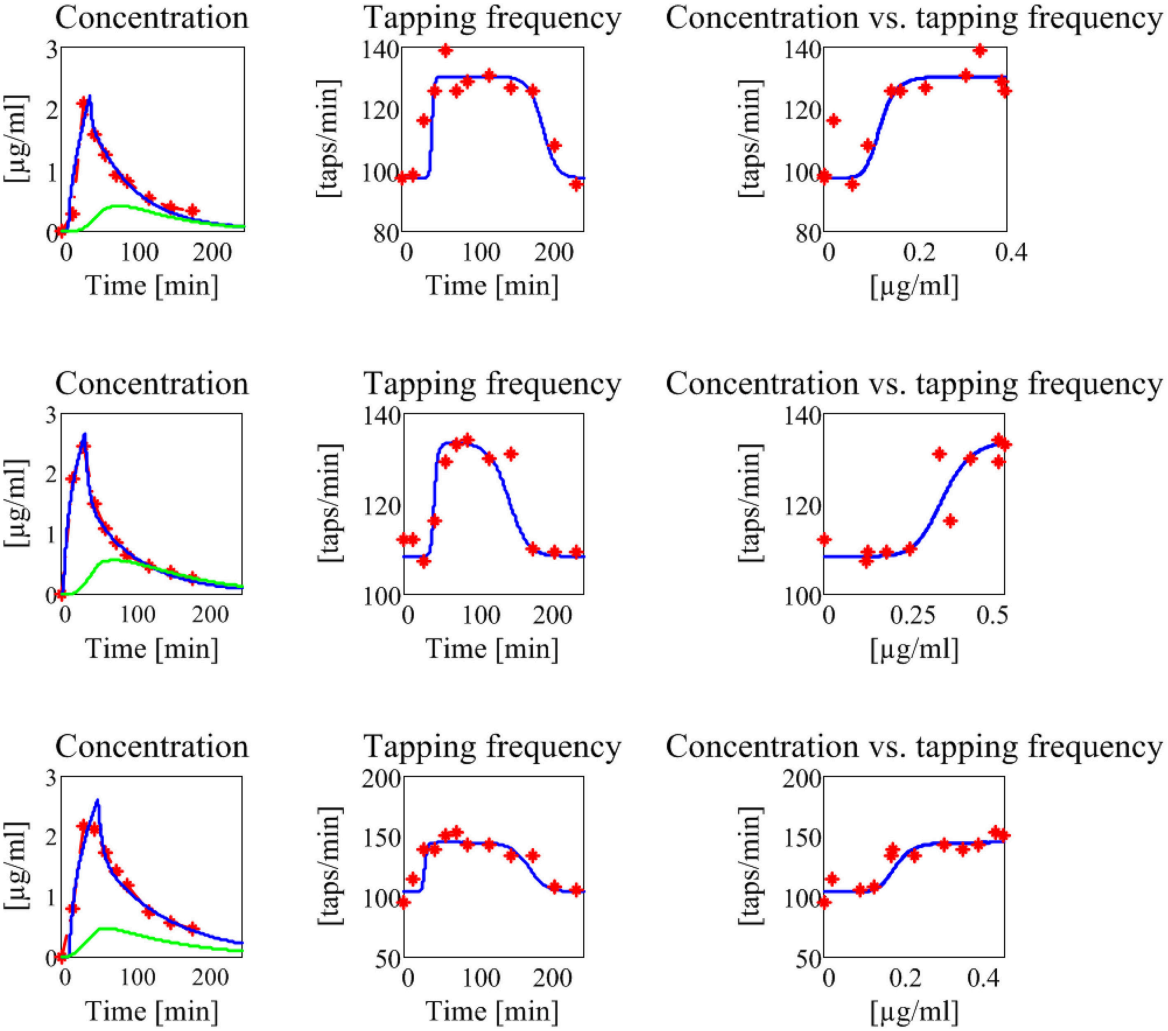
**Results concerning the three patients in the second group (subjects with no motor fluctuations)**. The meaning of all the plots, curves, and symbols is the same as in Figure 7.

The results are shown in Figure 7, for what concerns the three patients of the first group, and in Figure 8 for the patients of the second group. The values of all estimated parameters are reported in Table 2, for what concerns pharmacokinetics, and Table 3, for what concerns the drug effect.

**Table 2:** **Parameters of the compartmental model obtained using the Nelder-Mead algorithm for minimization and corresponding cost functions: *k*_21_, *k*_12_, and *k_etot_* are in [L/min]**.

**Group**	**Subject**	***K21***	***k12***	***k_etot_***	***F_val_***
1	1	9.11	10.0	0.80	0.376
	2	8.7	7.4	1.16	0.113
	3	1.07	1.75	0.45	0.448
2	1	3.53	4.50	0.65	0.418
	2	1.26	1.77	0.58	0.055
	3	1.12	1.57	0.43	0.235

V_*1*_ and V_*2*_ values were set a priori at 12 L and 32 L, respectively, corresponding to mean values typical of a 70 kg man, as done by Aoki et al. (2014). The last column represents the criterion function after minimization, i.e., the sum of the square errors.

**Table 3:** **Parameters of the effect model, i.e., the drug removal rate *k*_*e*3_/*V*_3_, the delay *T*, and the parameters of the Hill equation tuned in order to fit the alternate finger tapping frequency pattern in each subject**.

**Group**	**Subject**	***k*_*e*3_/*V*_3_**	***T***	***D*_0_**	***D_max_***	***D*_*c*50_**	***N***
1	1	0.01	15	0.29	0.50	0.25	2
	2	0.02	0	0.28	0.317	0.03	2
	3	0.025	0	0.22	0.305	0.12	2
2	1	0.03	15	0.27	0.304	0.13	7
	2	0.02	15	0.279	0.31	0.38	8
	3	0.035	0	0.275	0.333	0.20	8

The absorption rate (k_31_/V_3_) was maintained at the same value (0.01) for all patients. k_e3_ /V_3_ is in [min^-1^], T is in [min], D_c50_ is in [μg/ml], D_0_, D_max_, and N are dimensionless.

**Table 4:** **Parameter values of the basal ganglia computational model. The parameters refer to the complete description and equations of the computational model represented in the Appendix section**.

**Name**	**Value**
τ / τ_*L*_	24 [ms]/120 [ms]
*a*	4
*u*_0_	1
ϑ_*G*_	0.3
*I^E^*	1
*I^I^*	3
*I^H^*	1.25
α	1
β	−1
γ	−1
σ	0.1
ϑ_*PRE*_	0.5
ϑ_*POST*_	0.5

**Table 5:** **Synaptic values of the basal ganglia computational model. The synaptic values refer to the complete description and equations of the computational model represented in the Appendix section**.

**Name**	**Projection**	**Type**	**Values**
*L*	Inhibition	Extradiagonal matrix	*l_ij_* = −1.2
			*l* ≠ *j*
*W^CS^*	Excitation	Full matrix	$w_{ii}^{CS}=1.1;w_{\begin{array}{l} ij\\ i\neq j \end{array}}^{CS}=0.2$
*W^CT^*	Excitation	Diagonal matrix	$w_{ii}^{CT}=4$
*W^GC^*	Excitation	Diagonal matrix	$w_{ii}^{GC}=0.48$
*W^GS^*	Excitation	Full matrix	$w_{ii}^{GS}=0.9;w_{\begin{array}{l} ij\\ i\neq j \end{array}}^{GS}=0$
*W^NC^*	Excitation	Diagonal matrix	$w_{ii}^{NC}=1.08$
*W^NS^*	Excitation	Full matrix	$w_{ii}^{NS}=0.1;w_{\begin{array}{l} ij\\ i\neq j \end{array}}^{NS}=0$
*W^EN^*	Inhibition	Diagonal matrix	$w_{ii}^{EN}=-2.2$
*W^IE^*	Inhibition	Diagonal matrix	$w_{ii}^{IE}=-3$
*W^IG^*	Inhibition	Diagonal matrix	$w_{ii}^{IG}=-12$
*W^TC^*	Excitation	Diagonal matrix	$w_{ii}^{TC}=3$
*W^TI^*	Inhibition	Diagonal matrix	$w_{ii}^{TI}=-3$
*w^ESTN^*	Excitation	Scalar	$w^{ESTN}=1$
*w^ISTN^*	Excitation	Scalar	$w^{ISTN}=14$
*k^E^*	Excitation	Scalar	$k^{E}=7$
*W^STNE^*	Inhibition	Row vector	$w_{i}^{STN}=-1$
*w^GH^*	Inhibition	Scalar	$w^{GH}=-1$
*w^NH^*	Excitation	Scalar	$w^{NH}=1$

The model, with a suitable choice of parameters, is able to simulate the patterns of levodopa concentration in plasma and the tapping frequency in all patients. Looking at the curves and at the estimated parameter values, no significant differences can be found between the two groups for what concerns the plasma concentration of levodopa. Conversely, significant differences can be observed in the relationship between the effect compartment concentration and its effect on the striatal neurons.

In particular, the patients in the first group have a much lower value of the parameter *N* (as low as 2 for all three) whereas patients in the second group have much higher values of *N* (7 or 8). This means that the relationship is much steeper for patients in the second group, and therefore that even a moderate reduction in levodopa concentration can produce a quick reduction of the tapping frequency.

Another parameter that seems higher in patients of the second group is *D*_*c*50_, i.e., the levodopa concentration at half of the maximum effect. This implies that the effect of levodopa starts to be reduced at higher level, and higher concentrations values are necessary to obtain a sustained effect. However, differences in this parameter are less evident between the two groups.

A parameter that seems to have a certain role is also the drug removal rate from the effect compartment, i.e., *k*_*e*3_∕*V*_3_. This parameter is higher in the second group than in the first, indicating a greater reabsorption rate in more severe PD patients.

It is worth noting that patient 3 in the first group exhibits value of *D*_*c*50_ and of *k*_*e*3_∕*V*_3_ more similar to those of the second group, suggesting that this patient is borderline between the two groups.

## Discussion

In recent years, we developed a simple model of action selection in the basal ganglia, which incorporates the main physiological mechanisms acting in the BG and represents a good compromise between simplicity and accuracy (Baston and Ursino, 2015a). The most important aspect of that model was the reproduction, although in a simplified form, of the dopamine effect on the BG (in particular on the Go and NoGo neurons in the striatum) taking into account both the basic level (i.e., tonic dopamine) and phasic changes (peaks and dips during rewards or punishments). An important novelty was also the inclusion of the dopamine-acetylcholine balance on the striatum, which allowed a much better reproduction of the dopamine influence (especially during a fall in dopamine, potentiated by an excitation of the cholinergic system).

Previously, other neurocomputational models contributed to our understanding of both cognitive and motor deficits in PD. They mainly focused on the notion that reduced dopamine increases the activity and causes long-term potentiation in the indirect pathway of the BG, as observed by Wiecki and Frank (2010). These authors also state that this view can account for progressive motor degeneration as well as cognitive issues. Frank (2005) tried to explain cognitive tests results of medicated and unmedicated PD subjects, and particularly avoidance behavior in unmedicated PD on both motor and cognitive sides, using a computational framework (the LEABRA network) that, although incomplete (the hyperdirect pathway was actually lacking), is able to provide some explanations on clinical results. In a subsequent work the same authors included all the three main routes (direct, indirect, hyperdirect), using the same mathematical approach (Frank, 2006). The model by Moustafa and Gluck (2011) instead focused specifically on PD's issues in a peculiar cognitive task, the “weather prediction” task, pointing out a key role of prefrontal cortex dopamine in addition to striatal dopamine in affecting the action selection process. Stocco et al. (2011) described a model quite similar to the present, considering general interneurons as well. PD condition was simulated lesioning dopaminergic neurons. On the pure motor side, the influence of BG in the motor program selection functions and dysfunctions has been investigated by means of a spiking neural model (i.e., detailed neural description) by Humphries et al. (2006). Schroll et al. (2014) proposed a completely new viewpoint with respect to the majority of the computational models in literature, proposing that the motor impairments and pathway imbalances assessed in PD might also result from dysfunctional synaptic plasticity in the BG. In providing evidence of their original position, they used a detailed mathematical model, faithful to biological knowledge of BG, and different learning rules according to different BG structures driving the synaptic plasticity. In the motor domain, reaching has been studied, being a simple motor task: Magdoom et al., 2011 provided an original model, deviating from the classical Go/No Go model of the BG by adding an intermediate regime called the “explore regime,” used to control the stochasticity of action selection. PD was simulated reducing the dopamine level and affecting the indirect pathway.

Compared to other models, our computational model belongs to the class of those that describe neuron dynamics with more simple and compact equations, still remaining constrained by the neurobiological architecture, being able at the same time to simulate both motor and behavioral aspects. This may contribute to understanding the nature of the computation performed by entire brain regions. Hereafter, we will specifically compare our mathematical implementation, our novelties and results with previous computational models in literature. Ashby and Crossley (2011) also introduced cholinergic interneurons, but using a detailed mathematical approach and interpreting these neurons only as a “switch” for learning events. Stocco et al. (2011) considered general interneurons, but did not account for their differential effect on Go or NoGo neurons of the striatum. In fact, the authors state that their role is only to release inhibition on projection neurons when proper cortical inputs are detected, thus allowing the incoming cortical signals to be processed. Other models (Frank, 2006; Wiecki and Frank, 2010; Cavanagh et al., 2011) share some similarities with the present one, especially in the physiological knowledge incorporated, but use different mathematical representations. The main differences are observed between our model and the model by Magdoom et al. (2011), which uses a much more simplified view of the basal ganglia and network structure, recalling more of an actor-critic model. Other relevant models lack some BG pathways and/or structures, compared with ours (Frank, 2005; Moustafa and Gluck, 2011; Stocco et al., 2011; Schroll et al., 2012).

Going back to our model, previous preliminary simulations (Baston and Ursino, 2015b) endorsed the potential usefulness of the model in the study of PD patients, for whom the relationship between dopamine level and action selection in the BG plays a crucial role. In particular, we showed that a reduction in dopamine levels induces a slowdown in the action time (Baston and Ursino, 2015b). A subsequent necessary step toward model application is now to test its behavior on real data, to verify the capability to reproduce effective responses in PD patients with different chronic levels, and to point out the relationships with the underlying neural circuitry.

Accordingly, the objective of this work was to simulate patient responses during a simple test (the alternate finger tapping) routinely used in the clinical practice. Hence, a simple BG model with only two action channels seemed suitable to grasp the main aspects of the test.

The first simulations, performed by varying the tonic dopaminergic input from a normal level to a level leading to severe bradykinesia (Figures 4–6), confirmed the existence of a clear monotonic relationship between the input and the velocity of movement selection (quantified by means of the tapping frequency). An important contribution emerging from Figure 6 is that this relationship is quite flat at high values of the input (*D*>0.4 in Figure 6), indicating a stable behavior. This points out that even quite large fluctuations in the input induce only minor fluctuations in the action selection velocity, as is typical for a healthy subject. Conversely, at low values of *D* this relationship becomes quite steep: at this position, even small changes in *D* can cause large alterations in the action selection velocity. We think this is the zone more disabling for a PD patient.

With this scenario in mind, we exploited the neurocomputational BG model within a more comprehensive integrated model, to mimic the overall chain of events from levodopa administration to the alternate finger tapping response. Using this global model, we then simulated the behavior of six PD patients, providing individual estimation of the most influential parameters. Furthermore, to account for patients' variability, three patients were chosen from a first group, showing no motor fluctuations, and three from a second group, with motor fluctuations.

In building the global model, we used a classic representation of pharmacokinetics and pharmacodynamics, already used in previous studies (Sheiner et al., 1979; Contin et al., 2001; Chan et al., 2004). Indeed, these former compartment models were adequate to simulate levodopa temporal effects, hence we did not deem necessary to modify them.

Actually, the great novelty of the present work is in the connection between the pharmacodyamic compartment and the BG neurocomputational model (see Figure 1). In other words, the output of the pharmacodynamic model is not directly related with the tapping frequency, as done in previous papers, but is added to the dopaminergic input of the neural model, thus acting on the excitation/inhibition balance between the Go and No Go pathways. This balance, in turn, affects the tapping frequency.

We wish to emphasize that, in our model, the wearing-off of levodopa effect depends on two concomitant factors, while only the first is usually considered in previous models:

(i) the sigmoidicity of the concentration/effect relationship (Equation (6)), mainly influenced by parameters *D*_*c*50_ and *N*. A steep relationship (i.e., the Hill curve) means a large influence of small levodopa changes, hence a possible unstable behavior; a high value of *D*_*c*50_ indicates the need for high levodopa doses;
(ii) the position of the input quantity, *D*, on the global curve depicted in Figure 6. The latter aspect, which reflects the behavior of the neurocomputational model, depends, above all, on the dopaminergic basal value *D*_0_, which sets the central working point on this curve. A subject with a high basic dopamine works positioned in the high segment of this curve (like a healthy subject with physiological high *D* values), and therefore exhibits a stable behavior independently on fluctuations of the dopaminergic input. In fact, as we demonstrated in our previous work too (Baston and Ursino, 2015a), in this case the velocity of the action selection is almost unaffected by moderate fluctuation in the input *D*. Nevertheless, changes in *D* in these subjects (as occurring during rewards and punishments) can have a significant effect on synapse learning, by potentiating or depotentiating a synapse via Hebbian plasticity. On the other hand, a subject with a low basal value of *D* (as typical of PD subjects) works in the steep portion of the curve in Figure 6, hence he is naturally more prone to large fluctuations in the action selection velocity. This is the zone where therapeutic interventions are more effective, but also where instability is more easy to occur, and where the use of the model may provide significant benefits in future applications (as discussed in the last part of this session).

To elucidate these concepts, and look for model clinical use, in this work we undertook a preliminary model validation in a clinical setting by estimating parameters on six patients. We are aware that six patients are too few to attempt a critical analysis of the estimated parameter values. Indeed, the aim of this work was not to provide an exhaustive statistical investigation on parameter estimates (that would require a large dataset and an automatic estimation algorithm) but rather to show that the model is able to simulate the temporal patterns of the alternate finger tapping response in various patients, with potential clinical applications. Nevertheless, we can derive some preliminary indications, which of course require a subsequent confirm on a larger dataset.

An automatic procedure was chosen only for fitting the parameters in the compartment model. In fact, in this model, due to the regularity of plasma levodopa, the fitting was unique and reliable, quite independently on the initial guess. Conversely, an automatic fitting for the Hill parameters in the effect portion of the model was not appropriate, at least in the present initial work, since, due to the large noise on the experimental data, the obtained parameter values were crucially dependent on the initial guess and influenced by some outliers. Hence, we preferred a manual investigation in the parameter space. We are aware that our conclusions may be not unique, since other parameter combinations might produce quite similar results. In future works, with more data available, we will develop a constrained automatic fitting procedure, to solve the aforementioned problems.

Among the estimated parameters, three of them seem to have a more relevant role in discriminating between stable and wearing-off patients. The most important is the Hill coefficient *N*, which sets the slope of the sigmoidal curve (Equation (6)). Critical patients in the second group have higher values of *N*, suggesting a steeper relationship between levodopa and its effect (see also the last column in Figures 7, 8). This signifies that even a small change in levodopa concentration may induce the passage from saturation to the lower level.

Another parameter that seems to play a role is *D*_*c*50_, which represents the value at which levodopa exerts half of its maximal effect. The greater values found in two patients of the second group signify that higher doses of levodopa are required to have sustained beneficial effects.

Finally, the parameter *k*_*e*3_∕*V*_3_, which describes the levodopa removal from the effect compartment, is also of interest, setting the velocity at which concentration in the effect compartment decreases. Two patients of the second group have higher values.

We expect that the previous parameters may have a clinical impact. In particular, a high value of *N* and, although less important, a high removal rate mean that a patient may rapidly decay from quite a stable behavior (corresponding to a high value of *D* in our model) to a bradykinetic behavior. This is related with the duration of the tapping response and, more generally, with the length of clinical effects. A high value of parameter *D*_*c*50_ implicates a smaller impact of levodopa on the patients, hence the necessity of higher doses. Indeed, patients with wearing-off phenomena may require almost two-fold higher levodopa concentrations (Contin et al., 1993).

The present results, although obtained on a small number of cases, emphasize the potential model benefits, and agree reasonably well with those found in former papers, where the pharmacodynamic model was directly linked to the tapping frequency. For instance, Contin et al. (1993, 2001) observed that patients with wearing-off phenomena required almost two-fold higher levodopa concentration (a result related with the parameter *D*_*c*50_ in our model) and that regression toward a more unstable response to levodopa was associated with an increase in the sigmoidicity index *N*. Moreover, these authors found no clear role for the maximum magnitude effect (*D*_*max*_) with the progression of the disease. All these results agree with our observations, as evident looking at Table 3. It is worth noting that all the previous parameter changes have a substantial implication on the relationship between plasma levodopa concentration and its effect. Although plasma levodopa kinetics may remain substantially unchanged, the same pattern may produce shorter and more elusive effects in the advanced patients, being associated with a more rapid onset but also with a curtailed duration of motor response.

As also discussed in Contin et al. (1993, 1994) this knowledge may have strong practical effects. It might assist in designing a better dose regimen, for instance by reducing the dose of levodopa and increasing its frequency in more critical patients. A challenge for future works may be to find the optimal curve for levodopa administration, after the individual parameters have been estimated, in order to reduce the bradykinesia periods but maintaining levodopa to a minimum. In patients like those of the second group, for whom even a small fall in levodopa level may induce a large change in responsiveness, a model application may be to predict the moment when the wearing-off is starting. A further aspect that may be studied with the model in future work is dyskinesia, which may depend on an altered balance between Go and No Go pathways, hence on dopamine levels too. Finally, patients with a poor responsiveness may also exhibit an unbalance between reward and punishment during learning tasks. The latter aspect may also have a strong practical impact.

In conclusion, we claim that this approach is original and may have important benefits in future works. Specifically, once parameters have been estimated on the individual patient, the global model can be used not only to simulate results of the alternate finger tapping test, but also to investigate the patient's behavior in other conditions of clinical interest. For instance, an excessive response to levodopa (as occurring in patients with elevated Hill coefficient *N*) may excessively trigger the Go pathway, resulting in hyperkinesia when levodopa concentration is high, or in bradykinesia, with a delayed or absent response, when the levodopa concentration falls down. Future works may concentrate on these aspects, for instance also evaluating the impact of some external noise (which is always present both in the inputs and in the neuronal responses) on the action selection, or the effect on synapse plasticity during tasks which require learning, as a function of the estimated levodopa effect parameters.

## Author contributions

CB and MU wrote the paper, developed the model, and performed the simulations. MC, PC, and GC provided the patients' data and contributed to data analysis and to the interpretation of results.

### Conflict of interest statement

The authors declare that the research was conducted in the absence of any commercial or financial relationships that could be construed as a potential conflict of interest.

## Supplementary material

Supplementary Material presents the Appendix of the paper. The Appendix for this article describes in detail the Latest version of the Appendix can be found online at: http://journal.frontiersin.org/article/10.3389/fnhum.2016.00280
