# Supplementary material for: A Mathematical Model of Levodopa Medication Effect on Basal Ganglia in Parkinson's Disease: An Application to the Alternate Finger Tapping Task
Source: Front Hum Neurosci. 2016 Jun 17;10:280. doi: 10.3389/fnhum.2016.00280 (PMC4911387; doi:10.3389/fnhum.2016.00280)
Supplement: Supplementary file 1 [file Appendix.pdf]

APPENDIX

Mathematical description of the neurocomputational model

The mathematical description of the computational model is completed by the parameters reported in Table 4 and by the synaptic weights reported in Table 5.

A. Individual neuron dynamics

Let i be a post-synaptic neuron, receiving synapses w_{ij} from pre-synaptic neurons j, whose activity is y_j. The neuron can eventually also have additional inputs coming from external sources (not usually graphically represented): these are summarized in a single term I_i.

Synaptic and non-synaptic inputs to the post-synaptic neuron i converge in a single variable x_i. If there are N pre-synaptic neurons projecting to the post-synaptic neuron i, we can write:

$$x_i = \sum_{j=1}^{N} w_{ij} y_j + I_i \tag{7}$$

In order to mimic the cell membrane integrative process, the input x_i is transformed in a post-synaptic variable u_i, using a first order differential equation with time constant τ.

$$\tau \frac{du_i}{dt} = -u_i + x_i \tag{8}$$

Finally, a sigmoidal function ς computes the activity of the neuron i, y_i, from the output of the previous differential equation u_i.

$$y_i = \varsigma(u_i) \tag{9}$$

As in Baston and Ursino, 2015a, b, the sigmoidal function ς was implemented as

$$y_i = \frac{1}{1+e^{-a(u_i-u_0)}} \tag{10}$$

where a and u_0 are parameters which set the central slope and the central position of the sigmoid.

B. Network connectivity

The spatial position of individual neurons is described by the subscripts i, $i = 1, \dots, N$ for the majority of the layers (S, C, Go, NoGo, Gpe, Gpi), where N denotes the number of action channels,

i.e., the number of possible conflicting actions to be chosen. In this work we use $N = 2$, to represent the two possible actions of the alternate finger tapping test. The STN and the cholinergic interneurons ChI do not need subscripts since they are both represented in the model as single units.

To describe connectivity in the network, synapses are represented with two superscripts and two subscripts. The two subscripts indicate the position of the post-synaptic and pre-synaptic neurons respectively. Superscripts indicate the target layer (to which the presynaptic neuron i belongs) and the donor layer (where the pre-synaptic neuron j is located) respectively. The acronyms used to indicate the individual layers are S: sensory cortex; C: motor cortex; T: thalamus; G: Go; N: NoGo; I: Gpi; E: Gpe; H: cholinergic interneurons ChI; STN: sub-thalamic nucleus. Moreover, L is used to denote the dynamics of lateral inhibition in the cortex.

The nature of the synapses, portrayed in Figure 3 by arrows, is represented by the specific color of the projections: excitatory projections are represented in green, while inhibitory ones are represented in red. Lateral inhibition is portrayed in orange.

Among all the synaptic matrices and synaptic weights, we underline that a different denomination is used for k^E, since this projection does not connect single neurons, but informs the STN about the conflict within the cortex C, expressed by means of an energy function E.

Cortex

The first set of equations describes how the activity of the neurons in the cortex C is computed.

We can write for $i = 1, \ldots, N$:

$$\tau_L \frac{du_i^L}{dt} = -u_i^L + \sum_{\substack{j=1 \\ i \neq j}}^{N} l_{ij} y_j^C \quad (11)$$

$$\tau \frac{du_i^C}{dt} = -u_i^C + \sum_{j=1}^{N} w_{ij}^{CS} s_j + u_i^L + w_{ii}^{CT} y_i^T \quad (12)$$

$$y_i^C = \varsigma(u_i^C) \quad (13)$$

The previous equations can be explained as follows. Every neuron of the cortex C receives excitatory input from the whole stimulus S and an excitatory projection from the corresponding neuron in the thalamus. Moreover, it also receives and additional input u_i^L reflecting lateral inhibition from the other neurons in the cortex. The latter is characterized by a different time constant τ_L. If the neuron of the thalamus is active, the neuron of the cortex receives the positive feedback necessary to win the WTA selection.

Go part of the striatum

The second set of equations describes the activity of the neurons in the Go .

We can write for $i = 1, \dots, N$:

$$\tau \frac{du_i^G}{dt} = -u_i^G + \sum_{j=1}^{N} w_{ij}^{GS} s_j + w_{ii}^{GC} y_i^C + \alpha \cdot D \cdot \left(y_i^G - \vartheta_G\right) + w^{GH} y^H \quad (14)$$

$$y_i^G = \varsigma(u_i^G) \quad (15)$$

As for the cortex C, every neuron of the Go receives excitatory input from the whole stimulus S and an excitatory projection from the corresponding neuron of the cortex C, starting here the direct (or Go) pathway. In particular, it is worth noting that the array W^{GC} is diagonal, reflecting the separation among the two different action channels.

Dopamine and cholinergic interneuron activity (y^H) modulate the activity of each Go neuron. D here represents the overall contribution of basal tonic dopamine and dopamine derived from levodopa medication.

Dopamine is excitatory ($\alpha > 0$) if the Go activity is above a certain threshold (ϑ_G), inhibitory on the contrary: this mechanism realizes the contrast enhancement effect (Hernández-López et al., 1997).

The cholinergic interneurons are always inhibitory ($w^{GH} < 0$) to the Go instead.

Both dopamine and acetylcholine exert tonic and phasic effects on Go activity.

NoGo part of the striatum

The third set of equations describes the activity of the neurons in the NoGo.

We have for $i = 1, \dots, N$:

$$\tau \frac{du_i^N}{dt} = -u_i^N + \sum_{j=1}^{N} w_{ij}^{NS} s_j + w_{ii}^{NC} y_i^C + \beta \cdot D + w^{NH} y^H \tag{16}$$

$$y_i^N = \varsigma(u_i^N) \tag{17}$$

Just like the Go, also every neuron of the NoGo receives excitatory input from the whole stimulus S and excitatory projection from the corresponding neuron in the cortex C, starting here the indirect pathway instead (hence, the matrix W^{NC} is diagonal).

Dopamine and cholinergic interneuron (y^H) modulate the activity of each NoGo neuron as well, but in a different way. D has the same meaning as described in the section above.

Dopamine is always inhibitory ($\beta < 0$) to all the NoGo neurons, while the cholinergic interneurons provides excitation ($w^{NH} > 0$) to the NoGo. Both dopamine and acetylcholine exert tonic and phasic effects on the NoGo activity, in a specular way than in the previous Go case.

Globus pallidus pars externa

The fourth set of equations describes the activity of the neurons of the Gpe.

Equations are for $i = 1, \dots, N$:

$$\tau \frac{du_i^E}{dt} = -u_i^N + w_{ii}^{EN} y_i^N + w^{ESTN} y^{STN} + I^E \tag{18}$$

$$y_i^E = \varsigma(u_i^E) \tag{19}$$

Every neuron of the Gpe receives an excitatory projection from the corresponding neuron of the NoGo part of the striatum, continuing the indirect (or No Go) pathway, while the excitation (w^{ESTN}) from the STN is part of a feedback loop to control STN activity, as previously mentioned.

Every neuron is tonically active at rest, thanks to the external input (I^E).

Globus pallidus pars interna

The fifth set of equations describes the activity of the neurons in the Gpi.

Equations are for $i = 1, \dots, N$:

$$\tau \frac{du_i^I}{dt} = -u_i^I + w_{ii}^{IG} y_i^G + w_{ii}^{IE} y_i^E + w^{ISTN} y^{STN} + I^I \tag{20}$$

$$y_i^I = \varsigma(u_i^I) \tag{21}$$

Every neuron of the Gpi receives an inhibitory projection from the corresponding neuron of the Go part of the striatum ($w_{ii}^{IG} > 0$) continuing the direct pathway, and an inhibitory projection from the Gpe ($w_{ii}^{IE} < 0$), while the excitation ($w^{ISTN} > 0$) from the STN is part of the hyperdirect way. Indeed, the STN excites all the neurons of the Gpi, which in turns inhibit the corresponding neurons in the thalamus, thus braking any action selection.

Every neuron is tonically active at rest. In particular, the external input (I^I) overcomes the inhibitory input coming from the Gpe: that is the reason why, although the Gpe provides inhibition to the Gpi, the Gpi is active in the tonic state and inhibits the thalamus.

Sub-thalamic nucleus

The sixth set of equations describes the activity of the STN.

We can write since y^{STN} and u^{STN} are scalar variables:

$$\tau \frac{du^{STN}}{dt} = -u^{STN} + k^E E + \sum_{j=1}^{N} w_j^{STNE} y_j^E \tag{22}$$

with $E = \sum_{\substack{i=1 \\ i \neq j}}^{N} y_i^C y_j^C$ (23)

$$y^{STN} = \varsigma(u^{STN}) \tag{24}$$

The STN is connected to the cortex C, its activity however does not depend on a single neuron, but on the overall activity of C, sensed by means of an energy function E. The latter reflects the

conflict occurring in the cortex, i.e., it signals the presence of two (in other cases even more) cortical neurons simultaneously highly active. The higher the E, the higher the excitation of the STN. This is how the hyperdirect pathway starts.

The projection from the Gpe is part of the feedback loop to control STN activity, as previously said.

Thalamus

The seventh set of equations describes the activity of the neurons in the thalamus T.

We have for $i = 1, \ldots, \mathrm{N}$:

$$\tau \frac{du_i^T}{dt} = -u_i^T + w_{ii}^{TI} y_i^I + w_{ii}^{TC} y_i^C \tag{25}$$

$$y_i^T = \varsigma(u_i^T) \tag{26}$$

Every neuron of the thalamus receives an excitatory projection from the corresponding neuron of the cortex C, and an inhibitory projection from the corresponding neuron of the Gpi: the imbalance between the two determines whether the corresponding action is gated or not. It is worth noting that the excitation from the cortex to the thalamus realizes, together with the backward excitation from the thalamus to the cortex, a positive feedback loop, which is an essential part of the WTA cortical mechanism.

Every thalamic neuron is tonically silent at rest, as a consequence of the tonic activity at rest of the Gpi.

Cholinergic interneurons

The last set of equations describes the activity of the cholinergic interneurons ChI.

We have since y^H and u^H are scalar variables:

$$\tau \frac{du^H}{dt} = -u^H + I^H + \gamma \cdot D \tag{27}$$

$$y^H = \varsigma(u^H) \tag{28}$$

The cholinergic interneuron is inhibited ($\gamma < 0$) by dopamine (D), hence it is influenced both from tonic basal values and dopamine coming from levodopa medication.

The ChI is tonically active at rest (I^H).
